# Functional Analysis of *GhEXLB2* in Regulating Cotton Resistance to Verticillium Wilt

**DOI:** 10.3390/plants15111616

**Published:** 2026-05-25

**Authors:** Xuechi Li, Madad Allah, Xuehan Zhu, Junwei Wang, Ran Zhong, Jianting Feng, Haohua Chen, Manhong Wang, Fei Wang, Shandang Shi, Hongbin Li

**Affiliations:** 1 Key Laboratory of Oasis Town and Mountain-Basin System Ecology of Xinjiang Production and Construction Corps, Key Laboratory of Xinjiang Phytomedicine Resource and Utilization of Ministry of Education, College of Life Sciences, Shihezi University, Shihezi 832003, China; 18699318595@163.com (X.L.); madadnewchina@stu.shzu.edu.cn (M.A.); xuehan_zhu@foxmail.com (X.Z.); 18287018531@163.com (J.W.); 19180106050@163.com (R.Z.); jianting2023@stu.shzu.edu.cn (J.F.); chenhaohua2020@163.com (H.C.); feiw@shzu.edu.cn (F.W.); 2 Department of Poultry Science, Mississippi State University, Starkville, MS 39762, USA; mw2911@msstate.edu.cn

**Keywords:** upland cotton, expansin, *Verticillium dahliae*, disease resistance, plant hormone, transcription factor

## Abstract

Verticillium wilt (VW), caused by the soil-borne fungus *Verticillium dahliae*, is a major disease that markedly compromises both the yield and fiber quality of cotton. In this study, we explored the function and underlying mechanism of the cotton expansin gene *GhEXLB2* in response to VW infection. Expression profiling revealed that members of the *GhEXL* family exhibit distinct patterns across tissues and under various biotic and abiotic stresses. Notably, *GhEXLB2*, which encodes an extracellular protein, showed the strongest induction following *V. dahliae* challenge. Ectopic expression of *GhEXLB2* in *Arabidopsis thaliana* promoted root elongation and root hair formation, and was associated with improved resistance to the pathogen. In contrast, silencing *GhEXLB2* in cotton via virus-induced gene silencing (VIGS) led to pronounced vascular browning, increased pathogen recovery, and a lower level of disease resistance. In addition, RNA-seq profiling of *GhEXLB2*-silenced (VIGS) cotton plants revealed that most differentially expressed genes were enriched in pathways related to phytohormone signaling and plant–pathogen interactions, with salicylic acid (SA) signaling and WRKY transcription factors emerging as central regulatory components. Analysis of the *GhEXLB2* promoter further identified multiple *cis*-acting elements associated with stress and hormone responsiveness. When integrated with protein–protein interaction (PPI) prediction data, these results suggest that *GhEXLB2* may be modulated by a network of transcription factors and signaling pathways. Collectively, the evidence supports a positive association between *GhEXLB2* and VW resistance. This study provides a framework for understanding expansin functions in cotton defense against VW.

## 1. Introduction

Cotton (*Gossypium* spp.) is an economically indispensable cash crop worldwide, providing the main source of natural textile fiber as well as raw materials for food products, livestock feed, and bioenergy production. Among cultivated species, upland cotton contributes over 90% of worldwide production due to its high yield potential and wide environmental adaptability [1]. However, the progress of germplasm innovation and molecular breeding for disease resistance in upland cotton remains relatively slow in recent decades. Verticillium wilt (VW), a destructive vascular disease caused by the soil-borne phytopathogen *V. dahliae*, severely constrains cotton growth, development, and yield formation. The fungal hyphae penetrate root epidermal cells, colonize vascular bundles, and induce vascular browning and leaf wilting in cotton. In addition to this, *V. dahliae* may impair absorption of water and nutrients through release of plant toxins, which in some extreme instances causes death of plants and this results in the potential loss of yield of 10–30% annually and more than 50% in heavily infected fields [2]. The long-term survival of *V. dahliae* in soil and its complex pathogenic mechanism greatly limit the efficacy of chemical and agronomic control measures. Therefore, mining disease resistance genes and dissecting their regulatory mechanisms are the fundamental premise for breeding durable VW-resistant cotton cultivars and ensuring sustainable cotton industrial development.

Expansins were first identified as cell-wall-loosening proteins that promote cell expansion, growth and organ development. Beyond possessing developmental roles, emerging evidence indicates that expansins also act as key regulators in plant–pathogen interactions, playing dual roles either facilitating pathogen colonization or enhancing host defense [3,4]. Discovered firstly in cucumber hypocotyls, expansins possess intrinsic cell wall extension activity. Phylogenetic reconstruction enables the classification of this gene family into four distinct clades, namely EXPA (α-expansin), EXPB (β-expansin), EXLA (γ-expansin), and EXLB (δ-expansin). According to previous research members within the same subfamily exhibit relatively high consistency in terms of gene architecture, conserved functional domains, and cis-acting regulatory sequences [5]. Evolutionary analyses have demonstrated that the four subfamilies evolved under divergent selection pressure, with EXPA representing the youngest clade followed by sequential differentiation of other subfamilies; independent gene duplication events further drove functional divergence of paralogous genes in this family [6]. It is also observed that independent gene duplication processes have taken place in the family and resulted in functional differentiation of homologous genes [7].

Functionally, expansins widely participate in multiple developmental processes, including cell wall remodeling, organogenesis, and fruit ripening [8,9,10]. They also mediate plant adaptive responses to biotic and abiotic stresses, and profoundly shape root system architecture by modulating root hair initiation, primary root elongation, and lateral root formation [11,12]. For instance, *AtEXPA7* in *A. thaliana* is exclusively expressed in root hairs, and suppression of this gene results in a defective growth of rhizoid [13]. Moreover, *AcEXPA23*-OE in kiwifruit has been shown to promote lateral root development [14]. In contrast, *TaEXPB23*-OE has been shown to promote the initiation of lateral root primordia and enhance cortical cell development [15]. Similarly, *TaEXPA7-B* is proposed to regulate root system morphology by facilitating the development of secondary root primordia and cortical cellular structures [16].

In plant–pathogen interaction, expansins exhibit context-dependent dual regulatory roles. Certain expansins facilitate pathogen invasion: cucumber expansins promote colonization of arbuscular mycorrhizal fungi by loosening host cell walls [17]. Meanwhile, reduced expression of *LeEXPA5* prevents the relaxation of cell walls, which reduces the rate of parasitism by root-gall nematodes [18]. After *HaEXLB2* is upregulated by pathogenic bacteria, it inhibits the lignin synthesis pathway in plants, rendering the cell wall—a physical defense barrier—ineffective and increasing the susceptibility of *Helianthus annuus* to infection [19]. Conversely, host flora may initiate defensive reactions through modulating the activity of particular expansin components. Mutant lines of *Arabidopsis* lacking functional *EXLA2* exhibit enhanced tolerance against infection by *Botrytis cinerea* [20]; suppression of *EXPA4* transcription in tobacco plants can promote the accumulation of salicylic acid (SA) and enhance defensive capacity against tobacco mosaic virus [21]; inhibition of *BrEXLB1* expression significantly enhances plant resistance to clubroot disease [22]. In peanut, *EXLB8*-OE has been reported to trigger jasmonic acid (JA) and abscisic acid (ABA) signaling cascades, thereby enhancing resistance against *Sclerotinia sclerotiorum* [23]. Notably, expansin proteins can increase disease susceptibility but can also enhance growth; for example, rice plants *OsEXPA10*-OE are more prone to brown planthoppers [24]. *OsEXPA1*-OE, *OsEXPA5*-OE, and *OsEXPA10*-OE also lead to increased susceptibility to several prevalent rice disorders including bacterial blight, bacterial leaf streak, and fungal blast disease [24,25]. This indicates that *OsEXPA1* may function as a key regulatory hub involved in balancing the trade-off between plant growth and defense responses.

Genome-wide characterization revealed 72 expansin protein genes in cotton [26]. Existing investigations into gene function have predominantly concentrated on the contributions of EXPA and EXPB members during fiber formation and differentiation. A substantial set of expansin family members display strong transcriptional activity during fiber extension, and are closely correlated with the establishment of fiber quality characteristics. For example, *GhEXPA1* significantly increase fiber length and boll number [27]. *GhEXPA8* is able to enhance the fiber length and fineness [28], and *GhEXLB2* is a central gene in cotton drought tolerance response [29]. In contrast, existing studies largely overlook the function of the *EXLB* subfamily in cotton VW resistance. Up to now, the expression characteristics of *EXLB* members in response to *V. dahliae* infection, as well as their biological roles and underlying regulatory mechanisms in VW defense, remain poorly understood. There is still a lack of genetic evidence clarifying whether cotton *EXLB* members participate in the modulation of immune response and growth adaptation under *V. dahliae* stress.

As a representative member of the cotton *EXLB* subfamily, *GhEXLB2* is presumed to be a promising candidate gene involved in cotton–*V. dahliae* interaction based on its conserved domain characteristics and inducible expression pattern under pathogen challenge. Nevertheless, its exact biological function in VW resistance, as well as its potential role in regulating cell wall modification and defense gene transcription, still lacks systematic experimental validation. In the present study, we first characterized the expression pattern of *GhEXLB2* in upland cotton upon *V. dahliae* infection. We further verified its function in VW resistance via cotton endogenous gene silencing and heterologous overexpression in *A. thaliana*. In addition, we explored whether *GhEXLB2* mediates defense responses by modulating extracellular matrix components or activating the transcription of defense-related genes. This study aims to clarify the biological function of *GhEXLB2* in cotton VW resistance, fill the research gap of *EXLB* subfamily in disease immunity, and provide valuable gene resources and theoretical basis for molecular breeding of VW-resistant cotton varieties.

## 2. Results

### 2.1. Expression Profiles of Expansin Gene Family Members in Gossypium hirsutum

**Figure 1:**
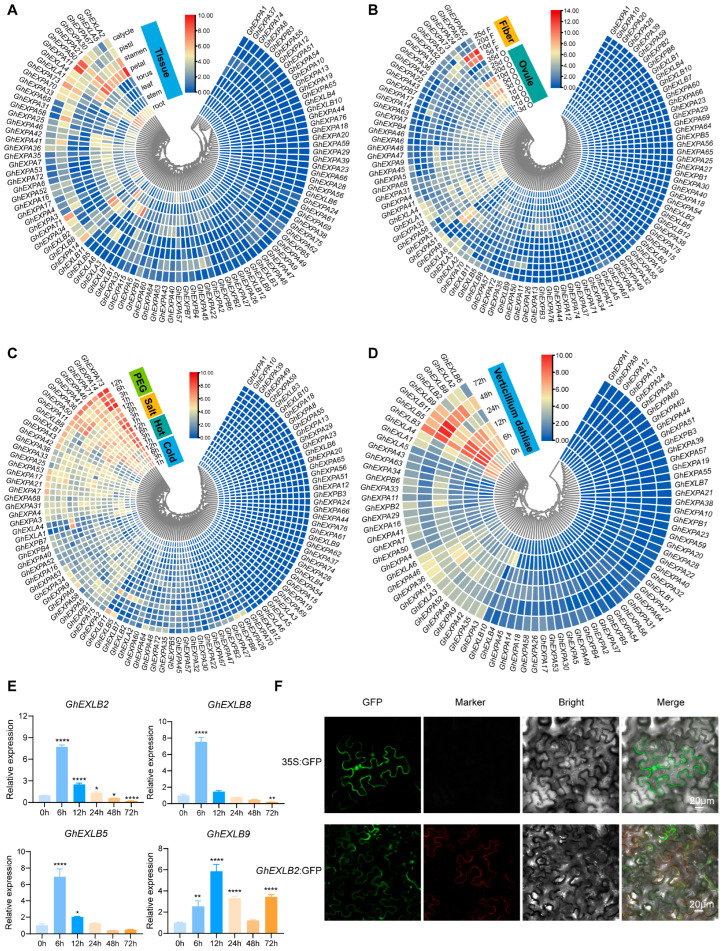
Expression features and subcellular localization of expansin family genes in upland cotton. (**A**) Expansin family gene expression patterns in various tissues during development. (**B**) Expansin family gene expression patterns in ovules and fibers during development. (**C**) Expansin family gene expression patterns under abiotic stress conditions. (**D**) Expansin family gene expression patterns under *V. dahliae* infection. Log_2_(FPKM + 1) values are represented by the color bar. (**E**) Quantitative real-time PCR (qRT-PCR) was used to assess the expression of expansin family genes in upland cotton under VW stress (n = 3). Statistical significance was indicated as follows: * *p* < 0.05; ** *p* < 0.01; **** *p* < 0.0001. (**F**) GhEXLB2 subcellular location. Scale bar = 20 µm.

A comprehensive spatiotemporal analysis of the expansin gene family in *Gossypium hirsutum* was performed to examine their expression across multiple tissues, pathogen infections, and environmental stresses to elucidate their transcriptomic patterns. Tissue expression profiling indicated that expansin genes showed higher expression levels in reproductive tissues compared with other plant organs, particularly high levels in calyces, pistils, stamens, and petals (Figure 1A). Conversely, their expression was relatively suppressed in non-reproductive tissues such as roots, stems, leaves, and receptacles, indicating an organ-biased expression profile with strong enrichment in bracts and pistils. During fiber development, the expansin genes exhibited high transcriptional activity in ovules at 0–10 DPA and fibers at 5–10 DPA. In contrast, transcript accumulation was markedly reduced in both fibers and ovules at the late stage (20–25 DPA) (Figure 1B). Under abiotic stress conditions, members of the EXPA and EXLB subfamilies were robustly induced. Specifically, 21 EXPA and two EXLB genes displayed elevated expression patterns under drought, salinity, heat, and cold stresses. Among these, *GhEXPA73*, *GhEXPA14*, *GhEXLB1*, and *GhEXLB8* were significantly upregulated as the stress duration increased, suggesting a pivotal role of the EXPA subfamily in mediating abiotic stress responses in cotton (Figure 1C).

Upon inoculation with *V. dahliae*, the EXLB subfamily was identified as the major responsive clade, implying its active involvement in cotton defense. Transcriptomic analysis revealed that EXLB genes exhibited maximum expression at 6, 12, and 48 h after inoculation, whereas relatively low transcript levels were observed at 0, 24, and 72 h post-inoculation (hpi) (Figure 1D). This expression pattern was further confirmed by qRT-PCR analysis of infected cotton roots, which showed that *GhEXLB2* reached its highest transcript level at 6 h post-inoculation, followed by a progressive decrease over time. In addition, expression at 6, 12, and 24 h remained significantly higher than that at 0 h (Figure 1E), leading to the selection of *GhEXLB2* as a candidate gene for further functional characterization of VW resistance. Subcellular localization prediction via CELLO suggested an extracellular role for *GhEXLB2*. To confirm this localization, a *35S-GhEXLB2–GFP* fusion construct was generated and transiently expressed in plant cells. Confocal imaging showed that *GhEXLB2–GFP* was observed in the extracellular region, consistent with apoplastic localization. Formal confirmation will require co-staining or plasmolysis in future studies. (Figure 1F). The subcellular localization results were consistent with the bioinformatics prediction, and we speculate that *GhEXLB2* may be involved in cell wall remodeling and extracellular defense.

### 2.2. GhEXLB2-OE Enhances Resistance of A. thaliana to V. dahliae

**Figure 2:**
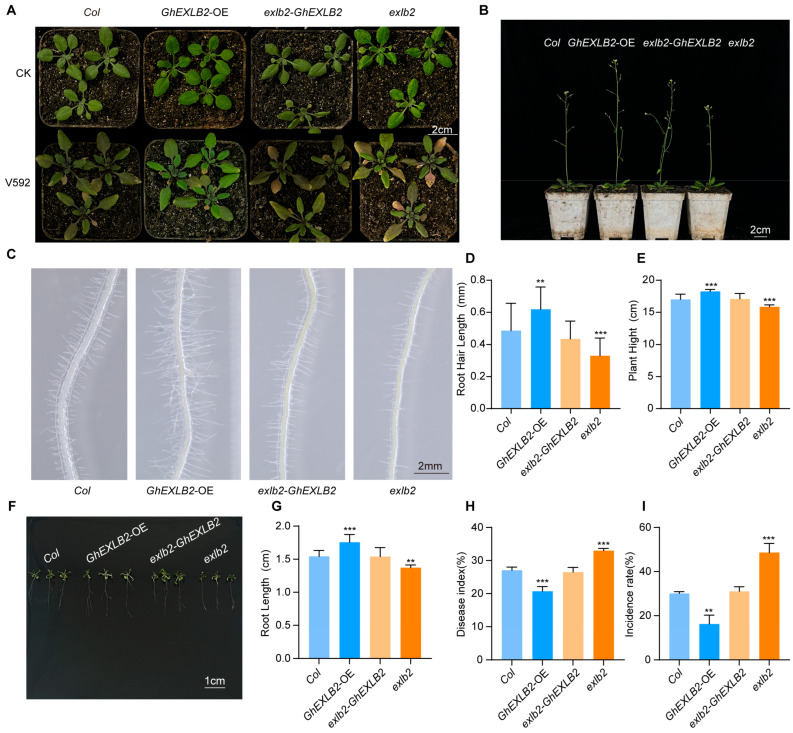
Phenotypic identification of *Col*, *GhEXLB2*-OE, *exlb2-GhEXLB2*, and *exlb2* mutant analysis following infection by *V. dahliae*. (**A**) Phenotypic variation in leaf phenotypes under natural conditions (water) and V592 infection. Scale bar = 2 cm. (**B**) Plant height comparison under standard growth conditions. Scale bar = 2 cm. (**C**) Root hair elongation under control conditions. Scale bar = 2 mm. (**D**) Statistics of root hair length comparison under normal growth conditions, (n ≥ 3). (**E**) Statistics of the plant height comparison in regular growth conditions, (n ≥ 3). (**F**) Root length comparison under normal conditions, (n ≥ 3); scale bar = 1 cm. (**G**) Root length comparison under normal growth conditions. (**H**) Disease index comparison following infection with *V. dahliae*. (**I**) Comparison of disease incidence after infection with *V. dahliae*. Data are shown as mean ± standard deviation (SD) from three independent experiments. Statistical significance is indicated as ** *p* < 0.01; *** *p* < 0.001.

To explore the biological function of the cotton *GhEXLB2* gene in plant defense against VW, we conducted functional verification experiments using transgenic *A. thaliana* materials. This study constructed a *GhEXLB2*-OE line, an *exlb2* mutant and *exlb2-GhEXLB2* line. Based on the controls of Columbia wild-type *A. thaliana* (*Col*) and the *exlb2* mutant, we performed the corresponding phenotypic analysis and *V. dahliae* infection study. Subsequent phenotypic observation revealed that the overall growth height of *GhEXLB2*-OE seedlings presented 7.3% (18.3 cm ± 0.3 vs. 17.0 cm ± 0.8) more than the plant height of *Col* under normal growth condition; the mutant was 6.8% (15.9 cm ± 0.3 vs. 17.0 cm ± 0.8) shorter than *Col*. Regarding root hair length, *GhEXLB2*-OE was 27.5% (0.6 mm ± 0.1 vs. 0.5 mm ± 0.2) longer than *Col*, while *Col* was 10.6% (0.4 mm ± 0.1 vs. 0.5 mm ± 0.2) longer than *exlb2-GhEXLB2*, and the *exlb2* mutant was 32.0% (0.3 mm ± 0.1 vs. 0.5 mm ± 0.2) shorter than *Col*. In addition, *GhEXLB2*-OE was 13.9% (1.8 cm ± 0.1 vs. 1.5 cm ± 0.1) longer in the taproot length than *Col*, the *exlb2-GhEXLB2* returned to the length of *Col*, and mutant was 11.2% (1.4 cm ± 0.0 vs. 1.5 cm ± 0.1) shorter than *Col* (Figure 2F,G). These findings suggest that *GhEXLB2* exerts a positive control over the plant height, root hair growth, and taproot in *A. thaliana*.

Under normal growth conditions (CK), no abnormal phenotype such as yellowing or wilting could be observed in the leaves of any of the lines. Following infection with *V. dahliae* (V592), however, mild yellowing of the leaves of the *GhEXLB2*-OE line was observed. The two lines exhibited leaf yellowing compared to wild-type *Col* and *exlb2-GhEXLB2* and the yellowing region of the *Col* was bigger and more severe. Phenotypic analysis revealed that the *exlb2* mutant exhibited reduced leaf size along with pronounced yellowing and wilting, indicating that loss of *GhEXLB2* markedly enhances the susceptibility of *A. thaliana* to *V. dahliae* (Figure 2A). With respect to plant height and lateral root development, the overexpression plants exhibited the greatest number of root hairs, while the mutant showed fewer and shorter root hairs. (Figure 2B,C). Quantitative assessment of disease resistance showed that, compared to *Col* plants, the *GhEXLB2*-OE line exhibited significant reductions in both disease index and incidence rate. Specifically, the disease index was decreased by 30.7% (from 27.0 ± 1.9 in *Col* to 20.7 ± 1.4 in the *GhEXLB2*-OE line), while the incidence rate was reduced by 46.2% (from 30.1 ± 0.8 to 16.2 ± 4.0) (Figure 2D,E). However, disease index and incidence rate of the *exlb2* mutant were found to be 28.5% (33.0 ± 0.7 vs. 27.1 ± 0.9) and 61.5% (48.6 ± 4.2 vs. 30.1 ± 0.8) higher than those of *Col* (Figure 2H,I). A combination of leaf phenotype, disease index, and incidence rate allows concluding that *GhEXLB2*-OE increases *A. thaliana* resistance to *V. dahliae* considerably and deletion of the gene results in reduced plant resistance. Finally, these results demonstrate that *GhEXLB2* not only significantly promotes plant height, taproot elongation, and root hair development in *Arabidopsis*, but also enhances resistance to *V. dahliae*, whereas gene deletion leads to weakened growth and significantly reduced disease resistance. *GhEXLB2* can promote root growth and development, so we cannot fully exclude the possibility that improved root architecture indirectly enhances plant disease resistance. Future research will further distinguish the roles of *GhEXLB2* in regulating growth and development from its immune and disease resistance functions.

### 2.3. Silencing of GhEXLB2 Gene Reduces Resistance to V. dahliae

**Figure 3:**
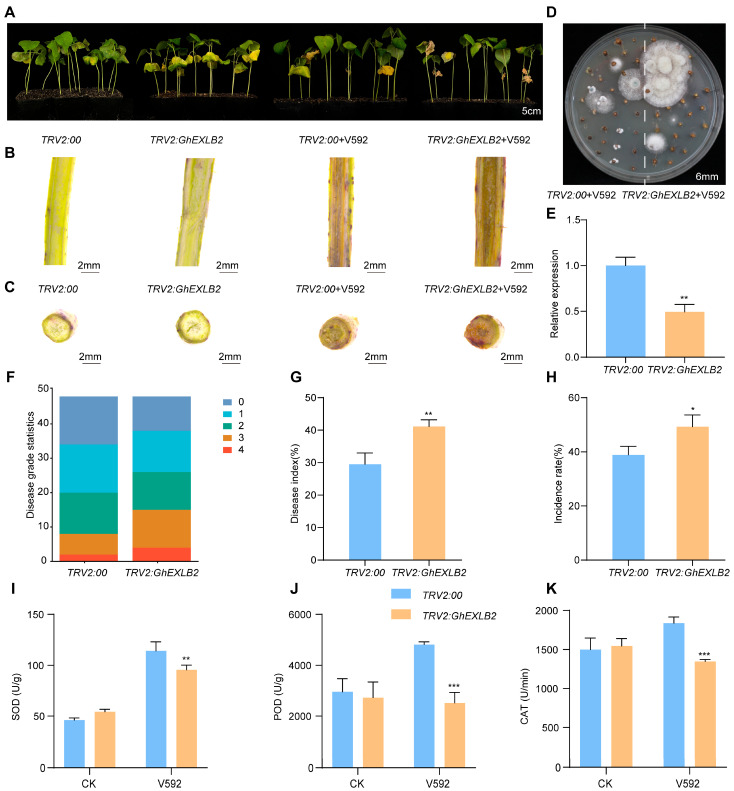
Phenotypic traits and physiological–biochemical parameters of *TRV2:00* control plants and *TRV2:GhEXLB2* plants were analyzed before and after infection with *V. dahliae*. (**A**) Phenotypic changes; scale bar = 5 cm. (**B**) Longitudinal section of stem, scale bar = 2 mm. (**C**) Transverse stem sections examined and post-inoculation with *V. dahliae*; scale bar = 2 mm. (**D**) Pathogen recovery from stems; scale bar = 6 mm. (**E**) Calculation of *TRV2:GhEXLB2* plant gene silencing efficiency. (**F**) Disease severity grading. (**G**) Disease index. (**H**) Disease incidence. (**I**–**K**) The effect of *TRV2:00* and *TRV2:GhEXLB2* on changes in SOD, POD, and CAT. Data are presented as mean ± standard deviation (SD) based on three independent experiments. Statistical significance is indicated as * *p* < 0.05; ** *p* < 0.01; *** *p* < 0.001.

To investigate whether *GhEXLB2* is involved in cotton defense responses against *V. dahliae*, this research prepared *GhEXLB2*-silenced cotton plants by using the VIGS technology. Plants carrying the empty vector construct (*TRV2:00*) were used as the control group. The phenotype, pathogen colonization, and physiological and biochemical indicators of plants infected with V592 were examined. The results are as follows: Prior to infection, *TRV2:00* and *TRV2:GhEXLB2* plants grew normal growth and did not show any noticeable difference. Plants that were infected with V592, *TRV2:00*, displayed slight yellowing and wilting of leaves, whereas *TRV2:GhEXLB2* plants had extreme leaf drop and stem browning (Figure 3A). Analysis of both longitudinal and cross-sections of the stem showed that *TRV2:GhEXLB2* plants exhibited a markedly greater extent of vascular tissue browning than the *TRV2:00* control plants (Figure 3B,C). This result indicates that silencing *GhEXLB2* exacerbates the disease phenotype upon *V. dahliae* infection. Re-isolation of the pathogen from stem tissues showed that *TRV2:GhEXLB2* plants yielded a significantly greater number of *V. dahliae* colonies compared with the *TRV2:00* control plants (Figure 3D), which indicates that the silencing of *GhEXLB2* gene facilitated the colonization and multiplication of the pathogen in the plant. qRT-PCR results showed that *GhEXLB2* expression in *TRV2:GhEXLB2* plants was reduced by approximately 50.6%. The conclusions are conservative and reflect partial loss of function. (Figure 3E). Disease grade statistics revealed that the proportion of plants with Grade 3 and Grade 4 disease symptoms increased markedly after gene silencing (Figure 3F). The disease index and incidence rate of *TRV2:GhEXLB2* plants were 39.4% (41.1 ± 2.1 vs. 29.5 ± 3.5) (Figure 3G) and 26.8% (49.3 ± 4.3 vs. 38.9 ± 3.2) (Figure 3H) higher than those of *TRV2:00* plants. Enzymatic assays were performed to verify that, under non-stressed conditions, the activities of SOD, POD, and CAT did not differ significantly between *TRV2:00* and *TRV2:GhEXLB2* plants. Following *V. dahliae* strain inoculation, in *TRV2:00* plants, the activities of SOD, POD, and CAT were markedly elevated, whereas *TRV2:GhEXLB2* plants exhibited reduced enzyme activities, decreasing by 16.3% for SOD, 48.3% for POD, and 26.7% for CAT (Figure 3I–K). Reduced SOD/POD/CAT activity correlates with *GhEXLB2* silencing. This may arise from attenuated defense signaling or more severe tissue damage.

These results imply that silencing *GhEXLB2* could partially inhibit the induction of antioxidant enzymes during pathogen infection, which may attenuate the plant’s capacity to alleviate oxidative damage.

### 2.4. Transcriptome Profiling of Cotton in Response to GhEXLB2 Silencing

**Figure 4:**
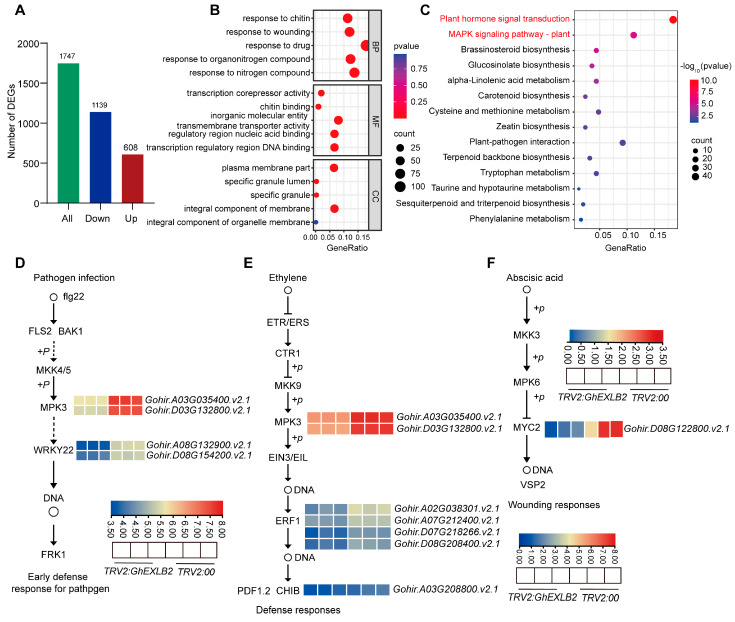
Transcriptome analysis of cotton plants upon *GhEXLB2* silencing. (**A**) Overview of DEGs identified in *GhEXLB2*–silenced cotton compared with control plants. (**B**) GO enrichment analysis of DEGs. (**C**) KEGG pathway enrichment analysis of DEGs, highlighting key defense and signaling-related pathways. (**D**) Expression profiles of genes involved in the pathogen-triggered early defense signaling pathway upon *GhEXLB2* silencing. (**E**) Expression changes in genes related to the ethylene signaling pathway after *GhEXLB2* silencing. (**F**) Gene expression patterns related to ABA and wound response pathways in cotton plants with *GhEXLB2* silencing.

To investigate the regulatory role of *GhEXLB2*, RNA-seq analysis was carried out on *TRV2:GhEXLB2* and *TRV2:00* cotton plants following *V. dahliae* treatment. In total, 1747 differentially expressed genes (DEGs) were detected, including 1139 genes with decreased expression and 608 genes with increased expression (Figure 4A). These observations indicate that silencing of *GhEXLB2* triggers extensive transcriptional reprogramming in cotton.

GO enrichment analysis (Figure 4B) revealed that the differentially expressed genes were highly enriched in immune and defense-associated biological processes, such as responses to chitin, mechanical damage, and nitrogen-containing compounds. Enriched molecular functions included transcription corepressor activity and chitin binding, while cellular component terms were mainly associated with membrane-related categories. These results imply that *GhEXLB2* contributes to membrane homeostasis and modulates the transcription of stress and immunity-related genes. KEGG pathway enrichment analysis (Figure 4C) showed that the DEGs were predominantly associated with plant hormone signal transduction, the MAPK signaling pathway, and α-linolenic acid metabolism, and plant–pathogen interaction. Among these pathways, plant–pathogen interaction displayed the most significant enrichment, supporting that *GhEXLB2* serves as a key regulator in the immune network against *V. dahliae*.

To validate the transcriptome data, we analyzed the expression profiles of key genes associated with major defense-related pathways (Figure 4D–F), including those involved in pathogen recognition (Figure 4D). *GhMPK3* and *GhWRKY22* were significantly upregulated in *GhEXLB2*-silenced plants. This suggests that gene silencing triggers activation of the MAPK–WRKY signaling module, thereby enhancing the expression of early defense-responsive genes. In the ethylene signaling pathway (Figure 4E), *GhMPK3* was induced, whereas the downstream *GhERF1* and defense marker genes *GhPDF1.2* and *GhCHIB* were downregulated. This suggests that *GhEXLB2* silencing affects ethylene-mediated defense responses through the MPK3-EIN3/EIL-ERF1 regulatory module. In the ABA and wounding response pathway (Figure 4F), *GhMYC2* was differentially expressed, and most wounding-related genes showed elevated expression levels. These findings reveal that *GhEXLB2* silencing triggers crosstalk between pathogen defense and wound repair pathways. Collectively, these data are consistent with the transcriptome and enrichment analyses, and further support that *GhEXLB2* functions as a central regulator that integrates MAPK, hormone, and stress signals to shape the immune response of cotton against *V. dahliae*.

### 2.5. Transcription Factor Expression and Protein–Protein Interaction Network Analysis Based on Transcriptomic Data

To gain insight into the regulatory framework associated with *GhEXLB2*-mediated disease resistance in cotton, we characterized transcription factor expression profiles from the identified DEGs, a PPI network was built, and the *cis*-regulatory elements present in the promoter region of *GhEXLB2* were further examined.

**Figure 5:**
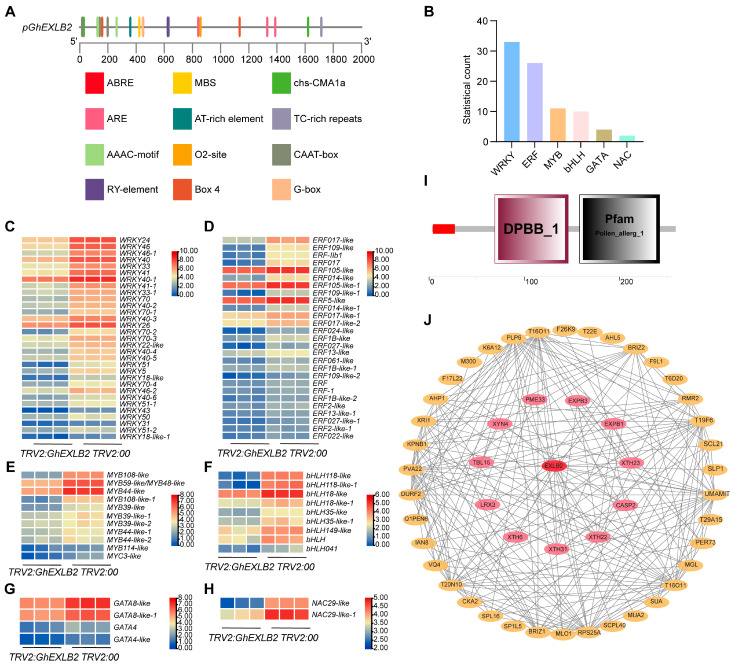
Analysis of transcription factor expression patterns and construction of a PPI network in cotton plants with *GhEXLB2* silencing. (**A**) *Cis*-regulatory element analysis of the 2000 bp putative promoter region upstream of *GhEXLB2*. (**B**) Statistical count of major transcription-factor-binding *cis*-elements in the *GhEXLB2* promoter. (**C**–**H**) Expression heatmaps of differentially expressed transcription factors from the WRKY (**C**), ERF (**D**), MYB (**E**), bHLH (**F**), GATA (**G**), and NAC (**H**) families in *TRV2:GhEXLB2* and *TRV2:00*. (**I**) Conserved domain analysis of GhEXLB2 protein. (**J**) PPI network using the STRING database.

A 2000 bp upstream region of the *GhEXLB2* coding sequence was retrieved and designated as the putative promoter for subsequent *cis*-element analysis (Figure 5A). A range of stress and hormone-responsive *cis*-elements were detected, including ABRE (ABA-responsive element), ARE (auxin-responsive element), and MBS (MYB-binding site), CAAT-box, and AT-rich regions. Statistical quantification showed that WRKY and ERF binding sites were the most abundant types of transcription factor recognition motifs, followed by MYB and bHLH elements (Figure 5B). These observations support the idea that transcription of *GhEXLB2* is coordinately modulated by diverse hormone signals and multiple transcription factor families, especially WRKY, ERF, and MYB.

Expression profiling of transcription factors revealed that the WRKY, ERF, and MYB families accounted for the majority of differentially expressed regulators (Figure 5C–H). Within the WRKY family (Figure 5C), several key defense-associated genes including *WRKY24*, *WRKY40*, *WRKY46*, and *WRKY41* were significantly downregulated in *GhEXLB2*-silenced plants relative to *TRV2:00*. For the ERF family (Figure 5D), ethylene-related genes including *ERF017*-like, *ERF109*-like, and *ERF22*-like showed reduced expression levels after silencing of *GhEXLB2*. In the MYB family (Figure 5E), *MYB44*-like and *MYB39*-like genes were notably repressed, and *MYC3*-like, a representative bHLH gene, showed a similar expression pattern (Figure 5F). Members of the GATA and NAC families also exhibited clear differential expression (Figure 5G,H). Among all these families, WRKY genes displayed the most prominent changes, implying that they serve as central regulators in the *GhEXLB2*-dependent transcriptional network. *Cis*-element analysis predicts potential regulation by WRKY, ERF, MYB, and ABA signals. Experimental validation (Y1H/EMSA/ChIP) will be required for confirmation.

The linear structural characteristics of GhEXLB2 were inferred through conserved domain analysis (Figure 5I). GhEXLB2 was predicted to contain an N-terminal signal peptide at its amino terminus supporting its potential role as a secreted extracellular protein. Two conserved functional domains were identified: the DPBB_1 domain together with the Pollen_allerg_1 domain, the latter corresponding to the expansin-specific Pfam domain PF01357. This domain organization is highly consistent with that of canonical EXLB subfamily proteins, reinforcing the functional involvement of *GhEXLB2* in cell wall remodeling and extracellular defense responses.

The STRING database was used to construct a predicted PPI network to explore potential functional associations among the DEGs (Figure 5J). The network identified a core regulatory module composed of transcriptional regulators (e.g., SKIP, TPL, MED15) and ribosomal proteins (e.g., RPL and RPS family members). These core components showed direct or indirect interactions with multiple disease resistance proteins (including CNGC, MLP, and LRR family proteins) and hormone signaling factors (e.g., AHP, ARR). *GhEXLB2* was located within this central module and interacted with cell-wall-modifying proteins (e.g., EXPB1, EXPB3, XTH family) and defense-related proteins (e.g., LRR, CASP2). Notably, the PPI network generated by STRING only provides computationally predicted functional associations, rather than experimentally verified physical interactions. These predictive results imply that silencing of *GhEXLB2* may reshape the overall protein interaction landscape, potentially coordinating transcriptional regulation, hormone signaling, cell wall remodeling, and immune pathways to modulate cotton defense against *V. dahliae*. Further experimental validation is still required to confirm the authentic protein–protein interactions and the underlying regulatory mechanism in future studies.

## 3. Discussion

The plant cell wall provides structural support for growth while also acting as the first physical defense barrier against pathogen attack, with its dynamic remodeling constituting an innate immunity [3]. Expansins have been widely investigated due to their roles in plant growth, development, and responses to environmental stress conditions [4]. In the present study, we preliminarily characterized the biological function of the expansin-like B gene *GhEXLB2* in upland cotton. Based on phenotypic evidence, we hypothesize that *GhEXLB2* may exert potential dual effects on vegetative growth and defense response against *V. dahliae*.

Upon *V. dahliae* infection, the transcription level of *GhEXLB2* was rapidly upregulated during the early infection stage (6–24 h) in cotton roots (Figure 1D,E), implying that *GhEXLB2* may participate in the early defense response of cotton. This indicates that *GhEXLB2* is one of the major components of the initial defense program of cotton. Following *V. dahliae* infection, the cotton *GhEG45* gene exhibited marked upregulation at 24 h and was subsequently characterized as a positive modulator of immune responses [30]. Subcellular localization assay clearly showed that GhEXLB2 protein accumulates specifically in the extracellular space (Figure 1F), further suggesting its potential involvement in extracellular defense processes. This suggests that *GhEXLB2* may be a component of the early defense response system in cotton. The *StEXLB1* gene is located in the cell wall and negatively regulates potato resistance to bacterial wilt disease [31]. *StEXPA* does not change their subcellular redistribution during pathogen interactions [32]. Combined with these findings, we speculate that extracellularly localized *GhEXLB2* may potentially participate in plant–pathogen interaction processes.

The growth and disease-resistant phenotypes of transgenic *Arabidopsis* and VIGS-silenced cotton plants provided intuitive phenotypic evidence for the biological function of *GhEXLB2*. Ectopic expression of *GhEXLB2* in *Arabidopsis* markedly enhanced plant height, primary (taproot) length, and root hair development (Figure 2B–G), and simultaneously alleviated disease symptoms after *V. dahliae* infection (Figure 2A,H,I). *GmEXLB1*-OE in soybean may enhance the *Arabidopsis* lateral root number and length [33], and other genes such as *AtEXPA7* in *Arabidopsis* and *AcEXPA23*-OE in kiwifruit have also been observed to enhance root growth [13,14,15,16]. VIGS-mediated downregulation of *GhEXLB2* resulted in a severely susceptible phenotype (Figure 3A–D). Meanwhile, the activities of antioxidant enzymes, including SOD, POD, and CAT, were notably lower than those observed in the control group (Figure 3I–K), indicating that the antioxidant capacity was relatively weakened after gene silencing.

Consistent with earlier studies, these results indicate that *AdEXLB8* confers stress resistance in plants through the induction of antioxidant defense mechanisms [23]. *OsEXPA10*-OE in rice actually predisposes the plant to brown planthopper and rice blast [34]. A large number of studies have shown that expansins and their associated genes play multiple roles in interactions between plants and pathogens. For instance, knockout of the *EXLA2* gene in *Arabidopsis* was shown to increase resistance against gray mold [20]. In resistant banana cultivars, infection by leaf spot disease was associated with reduced expression of *MaEXPLA6* [35]. Furthermore, *EXPA4*-OE in tobacco enhanced viral resistance [21]. Conversely, it is reported that silencing *GhEB1C* via VIGS significantly heightened plant susceptibility to Rhizopus [36]. These findings prove that *GhEXLB2* is a positive regulator that integrates root development and resistance to *V. dahliae* infection.

Resistance to VW in cotton is governed by a complex biological system that integrates multiple metabolites and signaling pathways. Key metabolites—including terpenoids, aldehydes, and phenylpropanoids—alongside phytohormones such as SA, JA, ethylene (ET), and gibberellins (GA), as well as reactive oxygen species (ROS), collectively contribute to this defense response [37]. The comparative transcriptomics data showed that *GhEXLB2* functions within a coherent disease resistance network by regulating multiple fundamental pathways. In *GhEXLB2*-silenced cotton plants, differentially expressed genes (DEGs) exhibited significant enrichment in major biological pathways, particularly plant–pathogen interactions and hormone signal transduction (Figure 4B,C). GO analysis enriched both the chitin response and fungal defense response. Meanwhile, KEGG pathway enrichment indicated a marked increase in activity within plant hormone signaling pathways as well as the plant MAPK cascade (Figure 4C). In the pathogen-associated pathway, key components mainly included the MAPK cascade (*MKK4/5*, *MPK3*) and the *WRKY22* transcription factor (Figure 4D). Following gene silencing, transcriptome data also showed that genes involved in ET and ABA signaling pathways were downregulated (Figure 4E,F), accompanied by decreased transcript levels of *ERF1* and *MYC2*. These transcriptomic alterations imply that *GhEXLB2* may potentially modulate VW resistance through ET- and ABA-dependent signaling pathways, although such regulatory relationships remain speculative. These results suggest that *GhEXLB2* may enhance cotton resistance to VW disease by regulating the ethylene and abscisic acid (ABA) signaling pathways. In *P. tremula*, treatment with ABA has been shown to alter the expression of specific expansin-related genes [38]; in tobacco, *AtEXPA18*-OE leads to elevated ABA levels in leaves [39]. *OfABL4* and *OfABL5* are likely to interact with the *OfEXLA1* promoter, enhancing its transcription in response to increased ABA levels, which in turn contributes to improved salt and drought tolerance in osmanthus while also supporting leaf growth [40]. Ethylene induces *AtEXPβ1* expression. *AtEXPβ1*-OX seedlings exhibit slender petioles [41], whereas ethylene exerts negative regulation on *AtEXPA5* expression, impairing lower embryonic axis growth [42]. This divergence indicates that different expansin protein members demonstrate diverse preferences in pathway selection during integrated hormone signaling. *AhEXLB8* is mostly promoted by the activation of JA and ABA pathways [23]. These studies collectively indicate that different expansin subfamily members may display diverse hormone response patterns.

The *GhEXLB2* promoter had several features of stress responses, including ABRE and ARE, and transcription-factor-binding sites, including MYB and ERF (Figure 5A). Suppressing *GhEXLB2* triggered altered expression patterns in several key transcription factors associated with plant defense responses, namely members of the WRKY, MYB, ERF, and NAC families (Figure 5C–H). PPI network prediction indicates that GhEXLB2 can interact with a number of cell wall modification proteins (Figure 5J). Importantly, all protein interactions predicted in this study are in silico predictions without experimental verification. Accordingly, we hypothesize that *GhEXLB2* may participate in cell wall remodeling, but this regulatory model requires further experimental validation. The process of actively fortifying its own walls by the use of its expansin-like protein is the opposite of the process of the nematode active secretion of the expansin-like *HaEXPB2* to loosen and weaken the plant cell walls [43,44]. Phytophthora capsici *PcEXLX2* is accidentally identified by plants and provokes immunity [45]. In contrast, the apoplast-localized *GhEXLB2* is speculated to modify cell wall structure in a defense-oriented manner. We tentatively propose a hypothetical model in which *GhEXLB2* may strengthen vascular physical barriers, limit pathogen colonization, and indirectly trigger downstream immune responses, including MAPK cascades and antioxidant systems. Nevertheless, all these mechanistic inferences remain speculative and need further experimental confirmation.

Based on the above phenotypic and transcriptomic evidence, we propose a preliminary working hypothesis to describe the potential function of *GhEXLB2* in cotton VW resistance. Under normal conditions, *GhEXLB2* may maintain cell wall plasticity. Upon *V. dahliae* infection, *GhEXLB2* expression is rapidly induced. We speculate that *GhEXLB2* may remodel cell wall components to enhance physical defense barriers and indirectly activate downstream immune cascades, including MAPK signaling and antioxidant defense. The differentially enriched hormone and MAPK pathways are considered potential downstream regulatory pathways, although some transcriptional changes may be secondary responses caused by aggravated disease symptoms. Additionally, *GhEXLB2* may indirectly modulate the expression of WRKY, ERF, MYB, and bHLH transcription factors via hormone signaling. Collectively, these potential molecular responses may restrict pathogen colonization and improve VW resistance. Notably, this study lacks direct biochemical evidence to confirm the regulatory relationships between *GhEXLB2* and hormone pathway genes. The downregulation of ABA and ET-related genes observed in transcriptome is regarded as an indirect correlative change. The association between *GhEXLB2* and the SA pathway is merely inferred from enrichment analysis. Therefore, the exact regulatory mechanism between *GhEXLB2* and hormone signaling remains unclear, and further experimental validation is required in future investigations.

## 4. Materials and Methods

### 4.1. Cultivation of Cotton, A. thaliana, and Tobacco, and Inoculation with V. dahliae

The cotton material in this experiment was upland cotton HuaXin103 and the cotton seeds were stored in our laboratory. HuaXin103 upland cotton seeds were soaked in water for 4–5 h. Plump seeds were chosen and planted in a mixed nutrient soil and grown in a 28 °C plant growth chamber under 16 h light/8 h dark photoperiod, under illumination of 80–200 μmol/m^2^/s.

*A. thaliana* ecotype Columbia (*Col*) was used in this study. Seeds were surface-sterilized using a unified procedure: seeds were soaked in five volumes of sterile ultrapure water, immersed in 75% ethanol, and then rinsed five times with sterile water. After sterilization, seeds were plated on half-strength MS agar medium, stratified at 4 °C for 48 h, and then incubated to a growth chamber at 20 ± 2 °C under a 16 h light/8 h dark photoperiod. Seedlings at the 3–4 true leaf stage were transplanted into mixed potting soil for further growth.

Tobacco seeds were sown directly in the same mixed soil and germinated in a growth chamber at 24 °C with a light intensity of 80–200 μmol/m^2^/s.

*V. dahliae* strain V592 was cultured on PDA medium at 28 °C for 5 days until full mycelial coverage. Mycelial agar plugs were collected using a sterile hole puncher in a laminar flow hood and inoculated into autoclaved Czapek liquid medium, followed by shaking incubation at 28 °C and 200 rpm for 7 days. Spore suspension was harvested by filtering through four layers of sterile gauze to remove mycelia. Spore concentration was determined using a hemocytometer and adjusted to 1 × 10^7^ spores/mL with sterile water for inoculation. Cotton seedlings at the two true leaf stage were inoculated using the root damage method: 5–10 mL of spore suspension was applied to wounded roots, and control seedlings were treated with an equal volume of sterile water. Inoculated seedlings were returned to the growth chamber, and root samples were collected at 0, 6, 12, 24, 48, and 72 h post-inoculation (hpi). All samples were quickly washed, frozen in liquid nitrogen, and stored at −80 °C until use. *A. thaliana* seedlings (25–30 days old) were gently uprooted and rinsed with tap water, then dipped in spore suspension for 1–2 min; control plants were treated with sterile water and recovered under standard growth conditions. Experimental replication: At least 15 plants per biological replicate were used for all inoculation assays. qRT-PCR and enzyme activity measurements were performed with three biological replicates and three technical replicates.

### 4.2. Genome-Wide Characterization of the Expansin Gene Family in Cotton

Genomic sequences from several cotton species were obtained from the CottonMD database, after which a local BLAST (TBtools, version 2.473) search database was established. The datasets included members of the AD genome group, such as *G. hirsutum* (JGI, AD1), as previously reported [46]. *A. thaliana* contains four subfamily genes of expansin, namely: EXPA, EXPB, EXLA, and EXLB [47]. Thirty-six protein sequences from *Arabidopsis* were retrieved from the TAIR database [48]. Homology-based searches of the upland cotton genome were conducted using BLASTP (TBtools, version 2.473) with the *Arabidopsis* expansin sequences as queries. To minimize false positives, the E-value threshold was established at 1 × 10^−20^. Candidate expansin genes obtained from the preliminary screening were further analyzed for conserved domains using the CDD database (http://www.ncbi.nlm.nih.gov/cdd/, accessed on 5 October 2025) [49]. Candidate proteins were confirmed as authentic expansins only if they contained the two canonical conserved domains—DPBB_1 (PF03330) and Pollen_allerg_1 (PF01357)—which are specific to the expansin superfamily. Following an assessment of sequence integrity, any redundant or fragmented sequences were discarded. Only sequences containing intact, expansin-specific conserved domains were finally confirmed and retained as authentic members of the cotton expansin gene family. The genes in the expansin family were referred to by their location in the chromosome.

### 4.3. Gene Expression Pattern Analysis and qRT-PCR Detection

PRJNA248163 [50] provided transcriptome data of various tissues under cold, heat, salt, and drought stress conditions. The laboratory upland cotton transcriptome database provided transcriptome data of the gene families during *V. dahliae* infection. Cotton roots were also used to isolate RNA using the total RNA extraction kit of plant (TIANGEB Biochemical, Beijing, China). RNA integrity was assessed using agarose gel electrophoresis. *GhUBQ7* was used as the internal reference gene, and qRT-PCR analysis was carried out with the Novozymes Q312 fluorescence quantification kit. Gene expression levels were determined using qRT-PCR with the same kit, and relative transcript abundance was calculated based on the comparative Ct (2^−ΔΔCt^) method. All primer sequences are provided in Supplementary Table S1.

### 4.4. GhEXLB2 Protein Subcellular Positioning

Primer sequences were designed using SnapGene v6.0.2, and the complete coding region of *GhEXLB2* was isolated via PCR amplification. After purification, the target fragment was integrated into the *pCAMBIA1300-GFP* backbone through homologous recombination. This process successfully yielded the recombinant fusion vector *35S:GhEXLB2-GFP* for subsequent functional assays. Primer information is provided in Supplementary Table S1. Competent cells of *Agrobacterium tumefaciens* GV3101 were transformed with the recombinant construct via a standard freeze–thaw protocol. The empty pCAMBIA1300-GFP and recombinant 35S:GhEXLB2-GFP vectors were separately cultured. After centrifugation, the bacterial pellet was collected and resuspended in a freshly prepared 10 mM MES, 10 mM MgCl_2_, and 1 mM AS, adjusting the final cell density to an OD_600_ of 0.8–1.0. Four- to five-week-old healthy tobacco plants were picked, and a blended bacterial suspension was then injected into the basal region of tobacco leaf tissues. The tobacco plants subjected to this bacterial infection were subsequently incubated in a dark environment for 1 day, and transferred to normal culture conditions for 48 h. GFP fluorescence was observed under a laser scanning confocal microscope with an excitation wavelength of 488 nm and an emission wavelength of 505–530 nm.

### 4.5. Virus-Induced Gene Silencing Experiment

A 300 bp silencing fragment for *GhEXLB2* was designed using the VIGS website (https://vigs.solgenomics.net/, accessed on 7 April 2025). The vector was constructed using the same method as described above. The recombinant silencing vector *TRV2:GhEXLB2*, positive control *TRV2:GhPDS*, empty vector *TRV2:00*, and helper vector *TRV1:00* were transformed into *A. tumefaciens* GV3101 using the freeze–thaw method. After positive verification, the verified *Agrobacterium* was inoculated into LB liquid medium supplemented with kanamycin (Kan) and rifampicin (Rif) and cultured to an OD_600_ of 0.6–0.8. The cells were then harvested through centrifugation and resuspended in infiltration buffer (10 mM MES, 10 mM MgCl_2_, 1 mM AS) to a final OD_600_ of 1.0–1.2.

Each resuspension of *TRV2:00*, *TRV2:GhPDS*, and *TRV2:GhEXLB2* was mixed with an equal volume of *TRV1:00* suspension and incubated in the darkness for 4 h. Cotton seedlings at the uniform two-cotyledon stage (before true leaf emergence) were selected for infiltration. The mixed suspensions were injected into the abaxial leaf surface of the seedlings. Injected seedlings were incubated in the darkness for 1 day and then transferred to normal growth conditions.

When the positive control seedlings (*TRV2:GhPDS*) showed a stable albino phenotype, newly developed roots from each treatment were collected for total RNA extraction. qRT-PCR was performed to evaluate the gene silencing efficiency. Seedlings with high silencing efficiency and negative control seedlings were inoculated with *V. dahliae* strain V592. The function of *GhEXLB2* in cotton resistance to VW was determined by measuring physiological and biochemical indexes and transcriptome analysis. All experimental data were analyzed using GraphPad Prism version 10.

### 4.6. Growing and Identifying of Transgenic A. thaliana Overexpression Lines

Plants of *A. thaliana* that were healthy (wild-type), having a growth cycle of about 3–4 weeks and homozygous mutant *A. thaliana exlb2* (SALK 034888C, the homozygosity of *A. thaliana exlb2* was measured using a three-primer method (Supplementary Materials contains the primers). Immature flower buds were opened prior to transformation and the recombinant plasmid *35S:GhEXLB2-GFP* in *Agrobacterium* GV3101 was introduced using the freeze–thaw method. LB liquid medium (Kan and Rif) was inoculated with the PCR-confirmed *Agrobacterium* and *Agrobacterium* grew until OD_600_ = 0.6–0.8, after which the bacteria were centrifuged. The bacteria were resuspended in 1/2 MS liquid medium, supplemented with Silwet L-77 to a final concentration of 0.02–0.03%, and the OD_600_ of the bacterial solution was adjusted to 0.6–0.8. The inflorescence of the plant was immersed into the bacterial suspension using the flower dip method [51] and swirled taking care not to push the plant deep into the suspension to achieve the maximum contact and enhance the efficiency of the transformation of the plant. Infiltration was conducted on a weekly basis for a total of three times. After each infiltration event, the plants were placed in dark and humid conditions for 24 h, then returned to normal conditions. Seeds from the T0 generation were collected for further screening. For selection, the T0 seeds were evenly distributed on 1/2 MS plates containing hygromycin at a concentration of 40 mg/L, then put under a vernal period of 2 days at 4 °C, and then put in a light incubator (20 ± 2 °C). Homozygous T3 transgenic lines were obtained through hygromycin resistance segregation analysis and further confirmed through qRT-PCR for stable *GhEXLB2* expression. Seedlings with dark green cotyledons and well-developed roots were selected and transplanted into nutrient soil. The positive plants were determined through the extraction of RNA and DNA. An internal control gene was *AtActin2*, and its expression was quantified using qRT-PCR (primers are presented in Table S1). Highly expressed plants were screened and T2 generation seeds were collected, and this process was continued until T3 generation. Subsequent experiments used homozygous T3 generation plants.

### 4.7. Disease Resistance Phenotype Analysis of Cotton GhEXLB2 Gene Silencing Plants and A. thaliana Plants

This paper evaluated the severity of the disease 15 days following inoculation on the extent of yellowing and wilting of the leaves. The severity of the disease was measured on a scale of 0–4, where Grades 0 represented healthy plants; Grade 1 reflected 1–2 affected cotyledons; Grade 2 reflected cotyledons, and Grade 3 indicated the involvement of two true leaves, whereas Grade 4 corresponded to the infection of all true leaves, withered or fallen off. According to the survey results regarding the disease grade, the disease index (DI) was calculated according to the following formula: DI = [Σ (Disease grade × Number of plants in that grade)]/(Total number of plants assessed × 4) × 100%. Disease incidence (I) was calculated as the percentage of affected plants or plant organs (e.g., leaves, ears) relative to the total number surveyed, using the formula: I (%) = (Number of diseased individuals or organs/Total number of individuals or organs assessed) × 100%. The disease index and disease incidence were calculated based on three independent biological replicates, and data are presented as mean ± standard deviation (SD). At 15 days post-inoculation, the colonization and browning of cotton stems were studied. Some of the cotton stems were put into the sterile tissue culture bottles and washed with sterile water. They were subsequently put on a clean bench, rewashed with sterile water five times, and of disinfected with 75% alcohol three times. They were then put through shaking in 10% NaClO at 37 °C and at 100 rpm for 10 min, washed with five additional washes with sterile water, disinfected with 75% alcohol, and finally washed with 3 additional volumes of sterile water. This procedure was performed 3 times. Subsequently, the disinfected stems were cut into about 1 cm pieces using a sterile scalpel and systematically placed on PDA plates with antibiotics (cephalosporin 200 mg/mL and streptomycin 100 mg/mL). The plates were allowed to dry in a laminar flow hood, then sealed and incubated in darkness at 28 °C for 5 days. The development of the pathogen was observed. For control, sterile ddH_2_O was applied to another set of stems. Stems of various plants were wiped and using a scalpel the same section of the stems were cut to get 1 cm long pieces of the stem that were then longitudinally cut open. The sliced stem segments were observed through a stereomicroscope (Leica, M165FC, Wetzlar, Germany) and photographed to document vascular browning.

### 4.8. Physiological and Biochemical Index Detection

Following the inoculation procedure described above, both *TRV2:GhEXLB2* and the *TRV2:00* were challenged with VW. To evaluate physiological and biochemical responses, the activities of SOD, POD, and CAT were measured in cotton root tissues. The assays were performed using reagent kits obtained from Beijing Solarbio Science & Technology Co., Ltd. (Beijing, China).

### 4.9. RNA-Seq and Subsequent Data Analysis

For transcriptomic analysis, root samples were collected from both *TRV2:00* and *TRV2:GhEXLB2* plants. After sampling, all tissues were immediately flash-frozen in liquid nitrogen and stored at −80 °C prior to downstream experiments. Total RNA extraction was carried out using IGENEBOOK Biotechnology Co., Ltd. (Wuhan, China), followed by high-throughput sequencing. Raw sequencing data were processed using FastQC and Fastp for quality assessment and adapter removal, generating clean reads for further analysis [52,53]. There were 3 biological replicates per group, generating ~6 GB clean data per sample, with Q30 > 96% and unique mapping rate of >90%. To align the clean reads, we employed HISAT2 against the reference genome of upland cotton cultivar HuaXin103 (JGI version), and FeatureCounts was used to count reads and normalize them to FPKM [54,55]. DEGs were determined using the R package edgeR, applying thresholds of false discovery rate (FDR) < 0.05 and |log_2_ fold change| ≥ 1 [56]. GO classification was used to functionally annotate the identified DEGs, while KEGG analysis was employed for pathway enrichment. PPI networks were then generated using the STRING database, and network topology was further analyzed with Cytoscape (https://cytoscape.org/, accessed on 1 March 2026).

### 4.10. Statistical Analysis

Statistical analyses were performed using GraphPad Prism. Differences among multiple groups were evaluated using one-way or two-way ANOVA, depending on the experimental design. One-way ANOVA was used for ≥3 groups, two-way ANOVA for time × treatment effects, and Tukey’s HSD post hoc test was used for multiple comparisons. Normality and variance homogeneity were confirmed. Exact tests are noted in all figure legends. Significance levels are shown in the figures as *p* < 0.05, *p* < 0.01, and *p* < 0.001, while *p* > 0.05 was regarded as not significant.

## 5. Conclusions

Here, we functionally characterized the expansin gene *GhEXLB2* from upland cotton and revealed its role in VW resistance. *GhEXLB2* acts as a positive regulator of root development and disease resistance, contributing to the balance between plant growth and immunity. Functional assays demonstrated that *GhEXLB2* significantly promotes root growth and enhances resistance to VW. Transcriptomic analysis further revealed that silencing of *GhEXLB2* impairs multiple defense pathways, including ethylene and ABA signaling, the MAPK cascade, and transcript levels of WRKY, ERF, and MYB transcription factors, all of which are essential for the full defense response against *V. dahliae*.

These findings uncover a novel role of *GhEXLB2* in integrating stress and immune signaling and provide valuable gene resources and theoretical support for cotton-disease-resistant molecular breeding.

## Data Availability

Data are contained within the article and Supplementary Materials. The raw transcriptome sequencing data have not yet been deposited into the Sequence Read Archive (SRA) database of the National Center for Biotechnology Information (NCBI). The data will be uploaded promptly upon manuscript acceptance, with the corresponding accession number and detailed metadata to be supplemented thereafter.

## Supplementary Materials

The following supporting information can be downloaded at: https://www.mdpi.com/article/10.3390/plants15111616/s1, Table S1: Primers used in this experiment.
