# Supplementary material for: Functional Analysis of GhEXLB2 in Regulating Cotton Resistance to Verticillium Wilt
Source: Plants (Basel). 2026 May 25;15(11):1616. doi: 10.3390/plants15111616 (PMC13259333; doi:10.3390/plants15111616)
Supplement: Supplementary file 1 [file plants-15-01616-s001.zip › plants-4318710-supplementary.pdf]

Supplementary Table S1: Primers used in this experiment

Primer name	Sequence (from 5′ to 3′)
UBQ7-F	GAAGGCATTCCACCTTGACCAAC
UBQ7-R	CTTGACCTTCTTCTTCTTGTGCTTG
QRT-*GhEXLB5*-F	ACTGCCTGCGGTGCTTGTTATC
QRT-*GhEXLB5*-R	GGTGTTGTCGCCTTCGCCATAG
QRT-*GhEXLB2*-F	TCATTCACGAGCAGCATACTACCC
QRT-*GhEXLB2*-R	AAGCACCACAACCAACACCATTG
QRT-*GhEXLB8*-F	GTGTTGGTTGTGGTGCTTGCTAC
QRT-*GhEXLB8*-R	AAGGCTCGTTGACTCAGGATGAAG
QRT-*GhEXLB9*-F	TCCTGACGGCTTAGGCACTCC
QRT-*GhEXLB9*-R	AGCAAGCACCACAGCCAATTCC
LP	GTGACGATGCTCGATCAATTG
RP	GCAAGGGATCCTTTGGTACTC
BP	ATTTTGCCGATTTCGGAAC
GhEXLB2-F	ATGGCTCTTTCTATTCAATCCCTT
GhEXLB2-R	GACATTAATTTGAACTCCTGTGTCA
1300-F	ttcgcaagacccttcctctatataag
1300-R	tgaacagctcctcgccct
1300-*GhEXLB2*-F	atttggagaggacagggtaccATGGCTCTTTCTATTCAATCCCTT
1300-G*hEXLB2*-R	ggtactagtgtcgactctagaGACATTAATTTGAACTCCTGTGTCA
AT-Actin-F	AACCACTATGTTCTCAGGTATCGCT
AT-Actin-R	GGGAAATGAAACAAACAAATGGAG
TRV1-F	TTCAATCCTTTGAACGCGGTAGA

TRV1-R	GCAAACCCATGAACCATGTTT
TRV2-F	GGTCAAGGTACGTAGTAGAG
TRV2-R	CGAGAATGTCAATCTCGTAG
GhPDS-F	GCCTGAAGACTGGAGAGAGAT
GhPDS-R	GCTTTACTCTGATCTGCAGATATTTCA
TRV2-*GhEXLB2*-F	aaggttaccgaattctctagaATGGCTCTTTCTATTCAATCCCTTT
TRV2-G*hEXLB2*-R	tgtcttcgggacatgcccgggTACAGTCACACCTTTGTCTGA
VIGS-QRT-*GhEXLB2*-F	AGGCGTGTTTCATGCAGCTA
VIGS-QRT-G*hEXLB2*-R	TCTCCATCCTCCCCACTCAA

Silencing fragment sequence of *GhEXLB2*：

ATGGCTCTTTCTATTCAATCCCTTTTGATCCTTTTCACAACTTTGCTTCTCAAAGGAACTCTGGTAGTGGCAGAAACATGCAGCAACTGTT
TCACTCATTCACGAGCAGCATACTACCCAAACTCTGACGAACAGGGAACAGATGTTGGCGCATGCGGTTTTGGTTCTTTTGGAGCAACA
ATAAATGGTGGAGATGTATCGGCAGTATCTGACCTCTACCGCAATGGTGTTGGTTGTGGTGCTTGCTACCAGGTGAGGTGCACCAACAG
CAACTATTGCTCAGACAAAGGTGTGACTGTA
